# Evaluation of chemopreventive potential of *Strobilanthes crispus* against colon cancer formation *in vitro* and *in vivo*

**DOI:** 10.1186/s12906-015-0926-7

**Published:** 2015-11-25

**Authors:** Nawal Al-Henhena, Shaden A. M. Khalifa, Rozaida Poh Yuen Ying, Salmah Ismail, Riad Hamadi, Abdrabu N. Shawter, Azila Mohd Idris, Ainnul Azizan, Nahla Saeed Al-Wajeeh, Mahmood Ameen Abdulla, Hesham R. El-Seedi

**Affiliations:** Department of Biomedical Science, Faculty of Medicine, University of Malaya, 50603 Kuala Lumpur, Malaysia; Department of Experimental Hematology, Karolinska University Hospital, SE-141 86 Stockholm, Sweden; Institute of Biological Science, Faculty of Science, University of Malaya, 50603 Kuala Lumpur, Malaysia; Department of Biochemistry, Faculty of Medicine, Sana’a University, Sana’a, Yemen; Department of Chemistry, Faculty of Science, University of Malaya, 50603 Kuala Lumpur, Malaysia; Division of Pharmacognosy, Department of Medicinal Chemistry, Uppsala University, Box 574, SE-75 123 Uppsala, Sweden

## Abstract

**Background:**

With cancer being one of the major causes of death around the world, studies are ongoing to find new chemotherapeutic leads. There are common mechanisms for colorectal cancer (CRC) formation. Several are connected with oxidative stress-induced cell apoptosis and others are related to imbalanced homeostasis or intake of drugs/toxins. Plants that have been used for decades in folk and traditional medicine have been accepted as one of the commonest sources of discovered natural agents of cancer chemotherapy and chemoprevention. The aim was to study the antioxidant and chemopreventive effects of *Strobilanthes crispus* on colorectal cancer formation.

**Methods:**

Five groups of rats were injected subcutaneously with AOM, 15 mg/kg body weight, each once weekly for 2 weeks. The cancer group was continued on 10 % Tween-20 feeding for 8 weeks. The standard drug group was continued on 35 mg/kg 5-fluorouracil intraperitoneal injection twice a week for 8 weeks, and the experimental groups were continued on 250 and 500 mg/kg *S. crispus* extract oral feeding for 8 weeks, respectively. The normal group was injected subcutaneously with normal saline once a week for 2 weeks, followed by oral administration of 10 % Tween-20 for 8 weeks. All the rats were sacrificed after 10 weeks. The colons were evaluated grossly and histopathologically for aberrant crypt foci (ACF). Gene expression was performed for *Bax*, *Bcl2*, *Defa24*, *Slc24a3*, and *APC* genes by real-time PCR. *S. crispus* and its fractions were evaluated for their chemopreventive effects against human colorectal adenocarcinoma cell line HT29 and cytotoxicity for normal human colon epithelial cell line CCD 841, and the active fraction was assessed for its components.

**Results:**

We observed significant decrease in total colonic ACF formation, malonaldehyde (MDA) and lactate dehydrogenase (LDH), increase in superoxide dismutase (SOD), up-regulation of *APC*, *Bax* and *Slc24a3*, and down-regulation of *Defa24* and *Bcl*-2 in rats treated with *Strobilanthes crispus*.

**Conclusion:**

Our results support the *in vivo* protection of *S. crispus* against CRC formation (azoxymethane-induced aberrant crypt foci) and suggest that the mechanism is highly specific to protect from oxidative insults and the following apoptotic cascade.

**Electronic supplementary material:**

The online version of this article (doi:10.1186/s12906-015-0926-7) contains supplementary material, which is available to authorized users.

## Background

It is without doubt that the imbalance between the production of excess free radicals and the ability of the cell to neutralize their proximate environment results in serious consequences such as damage of cell membrane, disruption of the cytoskeleton proteins harmony and deformability of the RNA and DNA components. The gastrointestinal tract is a very sophisticated and complicated organ, characterized by a high metabolic rate, enteric nervous plexus with numerous feedback loops, intermingling connections of various enzymes and hormones, and enormous turn-over of metabolites and toxins. Thus the gastrointestinal tract is highly susceptible to oxidative radicals and accordingly the incidence of mutations and genetic alterations [1]. The colorectal segment of the gastrointestinal tract is particularly vulnerable due to both the biological function and distal position. Hence, it is not surprising if colorectal cancer (CRC) accounts for 13 % of all cancers worldwide and considered as one of the world’s most common neoplasms [2]. Aberrant crypt foci (ACF) are one of the earliest neoplastic lesions of CRC and an evidential landmark during the early stage of tumour formation [3, 4].

Azoxymethane (AOM) induction has been proven to alter the metabolic balance of the intestinal epithelial layer, producing hydrogen peroxidase that in turn leads to thiobarbituric acid-reactive substances (TBARS) release and formation of free radicals [5]. Previous literature had documented the different aspects of oxidative damage attributed also to the up- and down-regulation of vital genes [6]. Adenomatous polyposis coli (*APC*), the pro-apoptotic Bcl-associated X (*Bax*) and anti-apoptotic B cell leukemia 2 (*Bcl*-*2*) genes are among the altered genes reported in CRC caused by oxidative stress [7]. Defensin, alpha, 24 (*Defa24*) and solute carrier family 26, member 3 (*Slc26a3*) are newly defined genes that have variant expression in colon tissues of AOM induced CRC [8]. The accumulating data support the notion of the role of oxidative stress as a key player in the pathology of cancer [9]. The mechanisms underlying the pathogenesis involve the rate of cell proliferation versus cell death and accordingly CRC development and progression [10]. The apoptotic pathways were also assumed to be consequent to the oxidative cascade. Apoptosis is a progressive cell loss with two possible pathways, i.e. intrinsic and extrinsic [11]. Under pathological conditions, nitric oxide and related free radicals are thought to play an important role in initiating both pathways. The excessive proliferation, accumulation of abnormal cells/foci and the defect of the apoptosis contributed to the consequences of eventual cancer formation. Cascade of irreversible processes attributes to profound structural and functional disruption leading to a second wave of gene mutation [12].

The same biological events can be mimicked *in vivo* and indirectly monitored *in vitro* to screen the protective effects of new chemotherapeutic agents. One of the oldest and well-known chemotherapeutic drugs, 5-fluorouracil (FU), is an anti-tumour agent broadly used in the management of compact tumours. FU inhibits thymidylate synthase (TS) and is incorporated into the RNA and DNA. However, FU has many side-effects and is cytotoxic to normal cells [13]. In this study we applied *in vitro* and *in vivo* strategy to study the chemopreventive effect of *Strobilanthes crispus*. Biological methods and genetic analysis were used to investigate the effects of *S. crispus* on AOM-induced ACF in rats and evaluate the gene expression in colon tissue. Traditionally, *S. crispus* is widely used as a laxative and to treat gastrointestinal tract disorders [14]. Its extracts has shown effectiveness against the hepatocarcinogenesis process [15]. It has been reported to protect the cell against oxidative insults [16] and possess antiproliferative [17], anticancer [18], gastroprotective [19], wound healing [20] and antimicrobial [21] properties. Several co-researchers have used traditional medicinal plants for the treatment of various elements including colon cancer [22]. The present work on *S. crispus* has revealed a potent protective effect against CRC formation in two-dimensional (*in vitro*) and three-dimensional (*in vivo*) horizons. The study was extended however to cover a broader range of extract fractions with biological examination and profiling in order to find new bioactive compounds that demonstrate an antioxidant and anti-proliferative biological action to develop anti-cancer drugs. This work is an extension of our continuous interest in isolation and identification of potent compounds from natural products [23, 24] and the goal was to test whether *S. crispus* can exhibit chemopreventive effect on colon cancer development *in vitro* and *in vivo*.

## Methods

### Preparation of plant extract

*S. crispus* was obtained from Ethno Resources Sdn. Bhd., Selangor, Malaysia, and identified using the voucher specimens deposited at the Herbarium of Rimba Ilmu, Institute of Biological Science, University of Malaya, Kuala Lumpur. Ethanol at 95 % was used to extract the plant for 3–4 days before filtration and low-pressure evaporation. For the animal study, the ethanol crude extract was dissolved in Tween-20 (10 % w/v).

### Experimental animals

Thirty healthy adult Sprague Dawley (SD) male rats (6–8 weeks old) were obtained from the Experimental Animal House. Each rat weighed between 150 and 180 g, and was placed individually in separate cages. The study was carried out in accordance with the approval of the Ethics Committee for Animal Experimentation, Faculty of Medicine, University of Malaya, Kuala Lumpur, Malaysia [Ethic No. PM/07/05/2012/MMA (b) (R)] and in agreement with the “Guide for the Care and Use of Laboratory Animals”, published by the National Academy of Science [25].

### Induction and chemoprevention of colon cancer in animals

Thirty male SD rats were divided into five groups of six rats each:

Group 1: (Placebo group) was injected subcutaneously with 0.9 % sterile normal saline once a week for 2 weeks and fed daily with 10 % Tween-20 (5 ml/kg) for 8 weeks.

Group 2: (AOM group) was injected subcutaneously with 15 mg/kg/ml of AOM once a week for 2 weeks and fed daily with 10 % Tween-20 (5 ml/kg) for 8 weeks.

Group 3: (FU group) was injected subcutaneously with 15 mg/kg/ml of AOM once a week for 2 weeks and injected intraperitoneally with 35 mg/kg 5-FU as standard drug twice a week for 8 weeks.

Groups 4 and 5: (*S. crispus* extract groups) were injected subcutaneously with 15 mg/kg/ml of AOM once a week for 2 weeks and orally fed with 250 and 500 mg/kg (5 ml/kg) *S. crispus* extract once daily for 8 weeks, respectively.

The experiment was terminated at the end of the 10 weeks, at which point the rats were anaesthetized with ketamine (50 mg/kg) and xylazine (5 mg/kg), and sacrificed.

AOM (Sigma Aldrich, St. Louis, MO, USA) was used to induce ACF in the rat colon after being dissolved in sterile 0.9 % normal saline. AOM was injected subcutaneously into the animals at a dosage of 15 mg/kg once a week for 2 weeks [26]. 5-FU (Calbiochem, USA) was used as a standard drug, used as intraperitoneal injection into the rats at a dosage of 35 mg/kg body weight twice a week for 8 weeks [27]. In our study, this particular dose was chosen based on IC_50_ values obtained from MTT tests. We used this concentration in the biological assays as it is in correspondence to the IC_50_ value for 48 h of treatment as previously described.

### Tissue collection

The colons were dissected, and slit open lengthwise from the anus to the rectum to evaluate the presence of ACF. Half of the colon tissue was fixed in 10 % phosphate buffered formalin, while the other half was placed in RNA solution (Ambion, Austin, Texas, USA) for further use [28]. For gene expression analysis, we selected *Bax*, *Bcl*-*2*, *Defa24*, *Slc24a3*, and *APC*.

### Gross quantification of colon mucosal ACF

Topographic analysis of the colonic mucosa was performed according to a published protocol [29] with slight modification. The colons were stained with 0.2 % methylene blue solution for 10 min and the total ACF number was determined then the multiplicity variables were used to quantify the aberrant crypts. The aberrant crypts were distinguished from the normal tissue by their increase in size, height, and discernible pericryptal zone. Aberrant crypt multiplicity was determined as the number of crypts in each focus (2 cm sections) and justified by the presence of four or more ACF.

### Histopathological examination

The colon was fixed for 24 h in 10 % buffered formalin solution for histological study. Under light microscopy, a scalpel blade was used to excise the ACF of interest and the surrounding normal crypts. A 2 × 2 mm dissected tissue was then sectioned (5 μm thickness), embedded and stained with haematoxylin and eosin (H & E).

### Estimation of antioxidant activity in colon tissue homogenate

Colon homogenates (10 % w/v) were prepared in cold 50 mM potassium phosphate buffer saline (PBS), pH 7.4. The cell debris was removed by centrifugation and the supernatant was used for the estimation of *in vivo* antioxidants using commercially available kits i.e. malondialdehyde (MDA) that indicates the level of thiobarbituric acid-reactive substances (TBARS; cat. # 10009055), and superoxide dismutase (SOD; cat. # 706002; Cayman Chemical Company, USA). All assays were performed according to the instruction manual of the manufacturer.

### Lactate dehydrogenase (LDH) determination

For LDH assessment, the blood samples were collected and separated for serum after complete clotting. The serum was assayed spectrophotometrically.

### Gene expression

#### RNA isolation and purification

The total RNA was extracted from 30 mg colon tissue in a highly denaturing guanidine-isothiocyanate-containing buffer using genomic DNA Eliminator columns combined with an RNeasy Plus Mini Kit (QIAGEN, Hilden, Germany). The total RNA concentration and quality were evaluated by determining the 260/280 absorbance ratio using a NanoDrop ND-2000 spectrophotometer (Thermo Fisher Scientific, Wilmington, DE, USA). The RNA samples were subjected to agarose gel electrophoresis (Additional file 1: Figure S1). The gel was examined after 30 min at 95 V under UV light to observe the 18S and 28S ribosomal RNA bands. The ratio of 28S RNA to 18S RNA was apparently 2:1.

#### Reverse transcription and cDNA synthesis

Complementary DNA (cDNA) was produced from 1 μg RNA of each sample using the ‘High Capacity RNA to cDNA’ master mix protocol (PN 4375575, Applied Biosystem, Foster City, CA, USA). The RNA was reverse transcribed to cDNA according to the manufacturer’s protocol instructions by adjusting RNA to 1 μg/20 μl with nuclease-free water and 4 μl RT (reverse transcription). The samples were loaded into a thermal cycler (Major Science, CA, USA).

#### Real time PCR normalization and amplification

The inventoried TaqMan gene expression assays were selected for the detection of rat RNA transcripts (Applied Biosystems, Foster, CA, USA). *Defa24* (Rn02769344_s1), *Slc26a3* (Rn00709709_ml), *APC* (Rn00560714_m1), *Bax* (Rn02532082_g1), and *Bcl-2* (Rn99999125_m1) were selected. *Hprt1* (Rn01527840_ml) and *Tbp* (Rn00560865_m1) were the endogenous control genes used for normalization in the rat colon tissue samples, following justification by geNorm and NormFinder. The analysis showed lower variability (low M value) of *Hprt1* and *Tbp*, which made them adequate for normalization. The PCR efficiency (E) for each gene and correlation coefficient (R^2^) was determined based on the slope of the standard curves generated using serial dilutions of the normal sample of 5-fold cDNA starting with 10 ng/μl. The efficiency was calculated and the accepted value was defined as being between 90 and 110 %. qRT-PCR was performed in triplicate in a total volume of 10 μl containing 5 μl of TaqMan fast advanced master mix, 0.5 μl TaqMan gene expression assay, 1 μl cDNA template, and 3.5 μl of nuclease free water. The average of the sample triplicate measurements was obtained for the threshold cycle (Ct) value. The comparative Ct (2-ΔΔCT) method was used to compare the gene expression of the targets. The relative gene expression was quantified as 2-ΔΔCT, where ΔΔCT = (ΔCT of treated sample RNA) – (ΔCT of untreated control or normal control RNA), and ΔCT = (CT target RNA) – (CT reference or endogenous RNA). The results were normalized to the reference genes and log^2^ to positive control [30, 31].

#### Crude extract fractionation

Firstly, column chromatography was performed to fractionate the plant extract in which a 2.0 × 50 cm glass cylinder column with packed silica gel 60 (0.063–0.200 mm, 70–230 mesh; Merck, Germany) was used according to a previously described method [32]. Different eluting solvents (hexane, ethyl acetate, methanol, acetone, acetonitrile, and water) were passed through the mixture of 1 g of plant extract/5 ml methanol to extract the fractions. The resulting fractions were collected, and an EYELA-L1 pump (Tokyo Rikakikai CO., Ltd., Tokyo, Japan) was used to ensure proper elution [28].

Secondly, thin layer chromatography (TLC) was performed, using aluminum foil pre-coated with silica 60 F254 plate (20 × 20 cm width and 0.2 cm thickness; Merck, Darmstadt, Germany). The TLC analysis was performed by using a mixture of ethyl acetate and ethanol and similar collected fractions were combined together resulting in six main fractions, denoted as STF1, STF2, STF3, STF4, STF5, and STF6.

#### Cell culture

The human epithelial colon cell line CCD 841 (ATCC® CRL-1790™) and colorectal adenocarcinoma cell line HT29 (ATCC® HTB-38™) was a gift from Department of Molecular Medicine, Faculty of Medicine, University of Malaya. They were grown in RPMI 1640 medium that was supplemented with 10 % (*v/v*) fetal bovine serum (FBS) (J R Scientific, Inc, USA), 1 % antibiotic solution (penicillin and streptomycin) (Sigma Aldrich, UK) and incubated at 37 ^o^C in a humidified atmosphere of 5 % CO_2_ in air [33]. The plant extracts and its fractions were prepared by dissolving in dimethyl sulfoxide (DMSO, Fisher Scientific, UK) at a concentration of 0.01 g/ml, and were serially diluted to 0.1–1000 μg/ml. The effect of the plant extract and its fractions on the cells were studied.

#### Testing the chemopreventive effects of plant extract fractions

The human epithelial colon cell line CCD 841 and colorectal adenocarcinoma cell line HT29 were seeded in 96-well plates at a density of 5 × 10^5^ viable cells/well and incubated for 24 h to allow cell adherence, then treated with plant extract and its fractions for 48 h. Subsequently, 10 μl MTT (5 gm/ml PBS) was added to each well and the cells were re-incubated for 4 h. The medium containing MTT was removed and 100 μl DMSO was added. Cell viability was determined spectrophotometrically at 595 nm. The fractions which showed positive results were further tested and identified by high performance liquid chromatography (HPLC) and liquid chromatography-mass spectrometry (LC/MS) to determine the cytotoxicity effects. Separation was achieved by using a Zorbax SB-C18 column (0.5 × 150 mm, 5 μm; part no: 5064-8256; Agilent, Vivantis company, USA) and the identification of bioactive compounds was detected using Agilent 6520 Accurate-Mass quadruple time-of-flight (Q-TOF) MS system via dual electrospray ionization.

#### Statistical analysis

Values were expressed as mean ± SEM. The variation between groups was estimated by the one-way ANOVA followed by Tukey’s post-hoc test using SPSS version 20 (SPSS Inc. Chicago, IL, USA). The real-time PCR data of gene expression were analysed using the GenEx Standard program version 6 (GenEx software, MultiD Analyses, Sweden). *T*-test was used to examine the differences between groups for all genes. *P* value of <0.05 was considered statistically significant. LC/MS data was processed by using the Agilent Mass Hunter Qualitative Analysis B.04.00.

## Results

### *S. crispus* extract inhibits formation of ACF

**Fig. 1:**
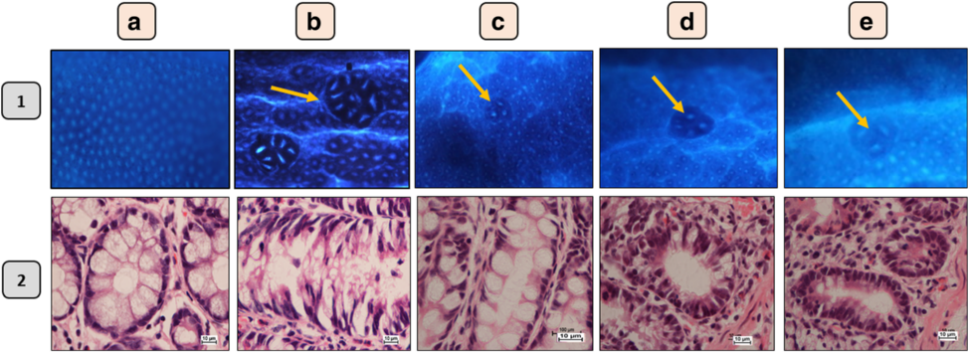
Effect of *S. crispus* on ACF. *1*- Rats colon tissue satin with methylene blue. *2*- Cross-section of the rat colon tissues stained with haematoxylin and eosin. **a** Normal group with normal, **b** AOM group, **c** 5-FU group, **d**: 250 mg/kg *S. crispus* group, **e**: 500 mg/kg *S. crispus* group. *1*-showed the crypts that were more than five in AOM group foci and less than five in all treated groups foci. *Arrows* indicated the crypts that were observed with methylene blue staining. *2*-Showed the elongated and slightly stratified nuclei found in AOM group and the depletion of mucin. Treated groups showed crypts with round nuclei which are normal

The occurrence and multiplicity of ACF in the colon were observed. The total number of ACF as well as the number of crypts per focus were counted. Multiple crypt clusters (more than four crypts/focus) of aberrant crypts per focus were also reported (Table 1). Rats treated with AOM and fed with either 250 or 500 mg/kg of the *S. crispus* extract showed a significantly lower number of total ACF/colon compared with the AOM-treated rats. The incidence of multiple aberrant crypts/focus was also significantly suppressed in rats fed with *S. crispus* extract as compared with the AOM-treated group. The inhibition of ACF formation as a marker for tumour initiation was recorded as 70.6–71.3 % in the *S. crispus* treated groups compared to 72.6 % in the fluorouracil-treated group when compared with the AOM group. The occurrence and multiplicity decreased dramatically in the *S. crispus* treated groups compared to both the positive and negative controls (Fig. 1-1).

**Table 1:** Effects of *S. crispus* extracts on the number of crypts per focus on AOM-induced ACF in rat colons

Group	Number of foci					ACF	
	1 crypt	2 crypts	3 crypts	4 crypts	>5 crypts	Total	Inhibition %
Cancer control group (AOM)	26 ± 7.15	36 ± 8.4	31 ± 4.4	25 ± 1.4	32 ± 7.3	150 ± 21.0	0
AOM + 5-FU	10 ± 2.1**	9 ± 2.3**	9 ± 2.6**	5 ± 1.5**	8 ± 2.6**	41 ± 9.6**	72.6 %
AOM+ *S. crispus* (250 mg/kg)	4 ± 1.4**	11 ± 2.3**	13 ± 0.5**	8 ± 2.5**	8 ± 2.4**	44 ± 4.6**	70.6 %
AOM+ *S. crispus* (500 mg/kg)	8 ± 0.5**	10 ± 2.7**	11 ± 2.1**	8 ± 1.2**	6 ± 1.2**	43 ± 2**	71.3 %

All values are expressed as mean ± SEM. **Significant difference at *p* < 0.01 (ANOVA, Tukey’s post hoc). *5-FU* 5-Fluorouracil, *ACF* aberrant crypt foci, *AOM* azoxymethane

### Histological analysis

**Fig. 2:**
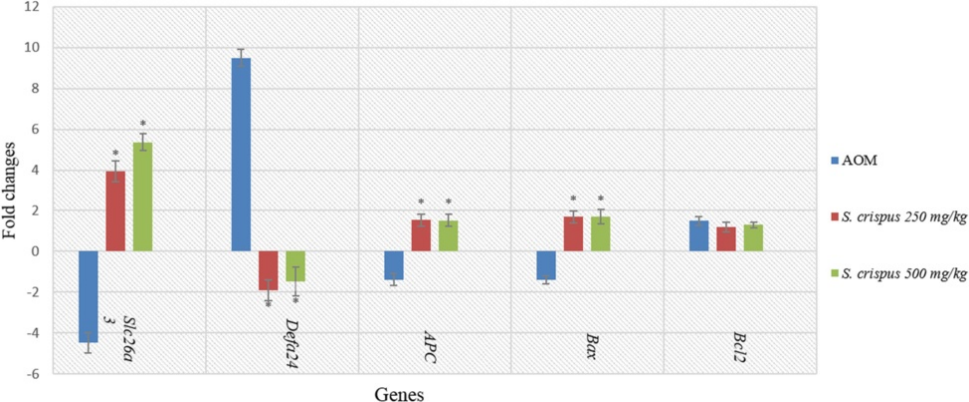
RT-PCR analyses showing changes in genes expression levels, expressed as log fold changes of *Slc6a3*, *Defa24*, *Apc*, *Bax*, and *Bcl2* genes of AOM and treated groups with *S. crispus* high and low dose (250 and 500 mg/kg) of plant extracts. Values were expressed as mean ± SEM. *Differences were significant at the 0.05 level vs. the AOM group

The large number of ACF in AOM rats was accompanied by enormous cellular changes. The stained crypts exhibited atypical nuclear organization, longer and larger mucosal lining, marked condensation of the nuclear materials and narrower lumen compared to the surrounding normal crypts. Moreover, the individual cells had distinctive elongated and stratified nuclei, loss of polarity, increase in mitoses, and lack of goblet cells (Fig. 1-2). Histopathological examination revealed the presence of proliferating mucosal glands with ACF characterized by elongated stratified nuclei and the depletion of mucin in the colonic tissue sections of AOM induced rats compared to the normal and plant treated rats.

### Effects of *S. crispus* on serum LDH and tissue MDA and SOD of colon homogenate

The group treated with AOM showed a high level of MDA and increased lipid peroxidation while it was markedly decreased in both *S. crispus* and FU treated groups. The reverse was seen with SOD, where elevated activity was observed with *S. crispus* but not in FU treated groups compared to the AOM group. *S. crispus* and FU treated groups showed significantly lower LDH activity compared to the AOM group (*p* < 0.01; Table 2).

**Table 2:** Effect of *S. crispus* on SOD and LDH activities and level of MDA in AOM induced rats

Groups	SOD (U/mL)	MDA (μM)	LDH (U/L)
Normal	6.17 ± 1.17	3.94 ± 0.27	1373.67 ± 137.29
AOM	8.16 ± 1.82	9.98 ± 0.78	1823.50 ± 97.96
FU	3.99 ± 1.12	3.60 ± 0.12***	776.33 ± 47.32**
*S. crispus* 250 mg/kg	10.98 ± 0.34*	4.34 ± 0.67***	729.17 ± 85.94**
*S. crispus* 500 mg/kg	11.21 ± 0.63*	3.21 ± 0.46***	779.67 ± 121.08**

All values are expressed as mean ± SEM. Significant difference at *p < 0.01, **p < 0.001, ***p < 0.0001 *vs* AOM group (ANOVA, Tukey’s). *FU* Fluorouracil, *AOM* azoxymethane, *LDH* lactate dehydrogenase, *SOD* superoxide dismutase, *MDA* malondialdehyde

### RT-PCR measurement

The endogenous genes *Hprt1* and *Tbp* were the endogenous control genes used for the normalization of the target gene expression (*Defa24*, *Slc26a3*, *APC*, *Bax* and *Bcl2*). The PCR efficiency (E) for each gene, including the endogenous control genes, was determined based on the slope of the standard curves which was between 94 and 156 %. The CT values were normalized to reference genes *Hprt1* and *Tbp* at slope between 3.2 and 3.5 and analysed by GenEx software. The analysis showed significant differences between the groups for all the target genes. The *APC* expression was up-regulated in the *S. crispus* groups with respect to the AOM group (Fig. 2). The expression of *Slc6a3* gene was significantly up-regulated by 3.957-fold and 5.373-fold, while *Defa24* was down-regulated by 1.897-fold and 1.456-fold in the low- and high-dose *S. crispus* groups, respectively, as compared to the AOM group (Table 3, Fig. 2). *Defa24* and *Bcl2* in turn were significantly up-regulated in the AOM group with respect to the placebo group, and *Slc26a3* was significantly down-regulated in the AOM group with respect to the placebo group. *Bax* was expressed in higher different ratios but *Bcl2* was expressed at a lower level in the extract groups with respect to the AOM group.

**Table 3:** Effect of *S. crispus* crude ethanol extract on gene expression of colon tissues of AOM-induced CRC in rats

Genes	*Slc26a3*	*Defa24*	*APC*	*Bax*	*Bcl2*
Animal groups					
Calibrator (Normal)	0	0	0	0	0
AOM	−4.47 ± 0.43^b^	9.49 ± 0.48^b^	−1.38 ± 0.31^a^	−1.40 ± 0.21^a^	1.49 ± 0.21^a^
*S. crispus* 250 mg/kg	3.96 ± 0.54^a^ ^b^	−1.90 ± 0.60^a^ ^b^	1.528 ± 0.34^b^	1.66 ± 0.29^b^	1.17 ± 0.24
*S. crispus* 500 mg/kg	5.37 ± 0.41^a^ ^b^	−1.46 ± 0.70^a^ ^b^	1.52 ± 0.33^b^	1.71 ± 0.35^b^	1.26 ± 0.16

The values were expressed as mean ± SEM, representing fold changes of target gene expression. Two-tailed unpaired student’s test was used. ^a^Significant difference at the 0.05 level between AOM-induced group and calibrator; ^b^significant difference between treated groups and induced (AOM) group

### Biological activity of the plant fractions and profiling of active compounds

**Fig. 3:**
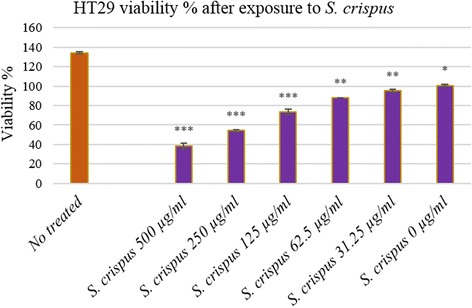
Effect of *S. crispus* on HT29 colon cancer cell line proliferation. Data were expressed as mean ± SEM for triplicates (*indicate the significant differences compared with control *P*-value ≤ 0.05 *P < 0.01, **P < 0.001, ***P < 0.0001 (ANOVA, Tukey’s)

**Fig. 4:**
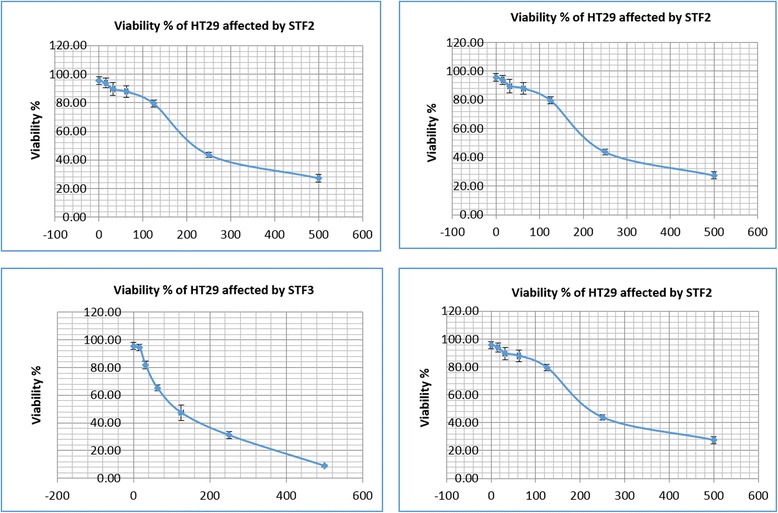
The effect of *S. crispus* fractions STF2 and STF3 on the viability of HT29 and CCD. Data were expressed as the mean ± SEM for triplicates Fig. 5: Effect of *S. crispus* fractions (STF2 & STF3) on HT29 colon cancer cell line proliferation. Data were expressed as mean ± SEM for triplicates

**Fig. 5:**
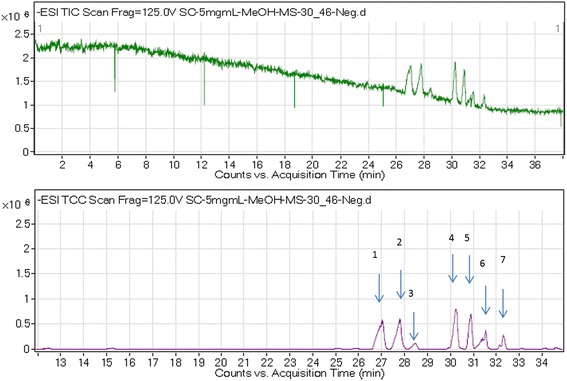
Chromatogram of STF3 of *S. crispus* negative mode (in 100 % methanol + 0.1 formic acid. The peaks numbers 1, 2, 3, 4, 7 represent the different RT and *m/z* of octadecadienoic acid, and peaks 5 and 6 represent urosolic acid and cis-12-oleic acid, respectively

**Fig. 6:**
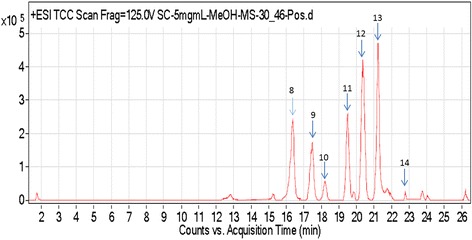
Chromatogram of SF3 of *S. crispus* positive mode (in 100 % methanol + 0.1 formic acid. The peaks numbers 8–14 represent ferulic acid, epigallocatechin gallate, lutein, icariin, triacylglycerol, caffeic acid and kaempferol, respectively

The fractions were assessed *in vitro* for their chemopreventive effects against human colorectal adenocarcinoma cell line HT29 and cytotoxicity for human colon epithelial cell line CCD 841. The crude extract that was assessed firstly against HT29 AND CCD481 (Fig. 3) was separated by column chromatography into six fractions (STF1-STF6) according to the differences in molecular size and polarity. They were investigated for their inhibitory effects against HT29 and CCD-841. The STF2 and STF3 of *S. crispus* at a concentration of 500 μg were the fractions that exhibited a significant inhibition value, and decreased the viability for HT29 cancerous colon cells to 27.43 and 9.09 %, respectively, with a corresponding low inhibition value on CCD-841 colon cells that increased the viability to 59.66 and 55.53 %, respectively. In contrast, low inhibition effects were observed when fractions STF1, STF4, STF5 and STF6 of *S. crispus* were used on the HT29 colon cancer cells. Fraction SF3 was further tested due to its potent activity by LC/MS and the peaks obtained were identified (Fig. 4). Caffeic and ferulic acids were isolated in addition to icariin, and epigallocatechin. Urosolic acid was also isolated from *S. crispus* (Figs. 5 and 6, Table 4).

**Table 4:** Identification of the major components of *S. crispus* SF3 by LC-MS positive and negative modes

Peak No.	RT	Suggested formula	Mass	*m/z*	Identified component
5	31.14	C30 H48 O3	456.36	455.35	Urosolic acid
8	16.35	C11 H16 O3	196.11	197.12	Ferulic acid
9	17.46	C22H18O11	452.34	453.34	Epigallocatechin gallate
11	19.49	C13 H20 O2	678.50	340.26	Icariin
13	21.27	C11 H16 O2	181.13	181.12	Caffeic acid

*RT* Retention time, *m/z* mass number (*m*)/charge number (*z*) of ions

## Discussion

*S. crispus* is a member of the Acanthaceae family. It is native to subtropical countries such as Madagascar, Indonesia and Malaysia. It is commonly known as ‘pecah beling’, ‘pokok pecah’, ‘pecah kaca’or ‘jin batu’ in Malaysia. Many active compounds have been identified from *S. crispus* with biological activities and pharmacological functions. In particular, verbascoside, glycosidic ester of caffeic acid and seven phenolic acids, namely *p*-hydroxy benzoic, *p*-coumaric, caffeic, vanilic, gentinic, ferulic, and syryngic acid have been isolated and identified from the leaves of the plant [34]. The leaves also contain a high amount of antioxidants, minerals and vitamins C, B1 and B2, as well as other flavonoid components, such as catechins, caffeine, and tannin [35]. Other co-researchers have studied the toxicity of *S. crispus* in SD rats, with no drug-related hazards, supporting the observations of this study [19, 20].

It was reported that plants belonging to the same family generally protect cells against oxidative insults and inhibit ROS formation in cell lines which ultimately cause cell death [36]. Distinct mechanisms of such protection were suggested, including increasing intracellular SOD activity. SOD catalyses the dismutation of superoxide into oxygen and hydrogen peroxide, thus protecting the cell from superoxide toxicity and harmful effects [36]. The high antioxidant activity of SOD has been reported as being an important factor in the treatment of inflammation of the colon in colitis and the inhibition of endothelial activation [37]. In addition, the decrease in the MDA levels leads to lowering of ROS levels, and redirects the metabolic pathway in correspondence with the presence of hydroxylated C3, unsaturated C ring, and hydrophobicity of the extract [17]. In this study, SOD activity was significantly elevated in *S. crispus* treated rats compared to untreated controls, underlining the antioxidant property of this plant.

We investigated the protective mechanisms of *S. crispus* ethanolic extracts on the morphology of intestinal crypts against carcinogenic changes induced by AOM. *S. crispus* ethanolic extracts rescued the architecture of the epithelial cells from damage, as seen by the minimum condensation of nuclear and cytoplasmic vacuoles, attenuation of early apoptosis, no decrease in the luminal space, and prevention of major changes in size and shape. *S. crispus* ethanolic extracts also counteracted the accumulation of ROS particles and thereafter overexpression of *Bax* gene, and increased the level of LDH in the animal serum [38]. In addition, *S. crispus* ethanolic extracts reduced the number of ACF in a dose and time dependent manner. Our results show that the protective effects of *S. crispus* ethanolic extracts are mediated, at least in part, by controlling the apoptotic pathway [39].

The protective effect of plant extract and its active constituents against cancer formation were examined genetically by using the real-time PCR method. The results of the present study showed that down-regulation of mRNA generated by mutation of the *APC* gene induced progression in CRC formation similar to that reported earlier [40]. Down-regulation of *APC* was associated with up-regulation of *Bcl-2* and increase in the mRNA level and thus prevented cells from undergoing apoptosis. This in turn induced cancer progression in the early stages of the genetic alteration cascades [41]. The *Bax* gene was down-regulated in this study; however, it was not statistically significant. The alteration of both *Bax* and *Bcl-2* gene expression was associated with improvement of the pathological state [42]. Consequently, the results suggested that deregulation of the apoptosis mechanism may have occurred [43].

Additionally, we investigated the newly-defined genes *Slc26a3c* and *Defa24* expression in colorectal tissue homogenates. It was found that *Defa24* appeared to be among the most up-regulated genes associated with AOM induced CRC, while *Slc26a3c* was among those that were down-regulated [8] indicating that the gene mapping was more stable under *S. crispus* ethanolic extract treatment. The other possibility is that *S. crispus* ethanolic extract protected protein conformation from undergoing a change that may be deleterious, which is propagated by free radicals at the genetic level.

We investigated the effects of exposure of two cell lines to the plant extract and its fractions, to determine the potential protective mechanisms of compounds in cancer formation. Exposure of HT29 and CCD-841 to plant extract and fractions induced a concentration-dependent decrease in cell viability as determined by the MTT assay. Apoptosis of HT29 and CCD-841 was indicated through the expression of apoptotic and antiapoptotic markers of *Bax* and *Bcl-2* genes. *S. crispus* fractions STF2 and STF3 displayed significant effect on HT29 cell death at 27.43 and 9.09 %, respectively. These two fractions were also effective on CCD-841 colon cells with viability of 59.66 and 55.53 %, respectively. On the other hand, the other four *S. crispus* fractions STF1, STF4, STF5 and STF6 were less effective on both cell lines. The major compounds identified were icariin and epigallocatechin, belonging to the flavonoid family of structures. These findings are in agreement with the literature describing *S. crispus* chemical composition [35]. Flavonoids have been suggested to have an effective role in the inhibition of carcinogenesis. Previous studies have reported the potency of flavonoids as antioxidants and their chemopreventive activity against many forms of cancer. Most of these studies have been carried out by using natural sources of flavonoids to test their antiproliferative activity on animal models [44]. Furthermore, some studies have reported the anticancer effect through in vitro studies [45]. In this study, different constituents of *S. crispus* showed different effects on the cells. The total flavonoid components of *S. crispus* (a mixture of flavonoids, caffeic, ferulic acids and urosolic acid) protected colorectal cells from oxidative damage and apoptosis. Flavonoids and other active compounds did show a synergic effect in the *in vivo* version of the experimental set. *S. crispus* as a whole and its components showed significant decrease in total colonic ACF formation, increase in SOD activity, significant decrease in LDH activity and MDA level, up-regulation of *Apc* and *Bax*, up-regulation of *Slc24a3*, and down-regulation of both *Defa24* and *Bcl-2*. Taken together, *S. crispus* and its composition of polyphenolic compounds demonstrated chemopreventive properties against CRC both *in vitro* and *in vivo*.

## Conclusion

Our study demonstrated the cytoprotective activity of S. crispus against zoxymethane-induced aberrant crypt foci. The in vitro and in vivo results supported the biological effect for a wide range of S. crispus extract fractions and extended to elucidate the underlying mechanism of action. The genetic profiling pinpointedthat the potent antioxidant effect was due to regulation of the genes involved in the apoptotic cascadederived from azoxymethane oxidative insult.

## Additional file

Additional file 1: Figure S1. Ethidium bromide-stained agarose gel samples that showed the extracted colon tissues RNA integrity (1) Seen under UV light. (2) Seen under Lourmat gel documentation system. (TIFF 256 kb)
