# Supplementary figures and images for: Evaluation of chemopreventive potential of Strobilanthes crispus against colon cancer formation in vitro and in vivo
Source: BMC Complement Altern Med. 2015 Nov 25;15:419. doi: 10.1186/s12906-015-0926-7 (PMC4658747; doi:10.1186/s12906-015-0926-7)

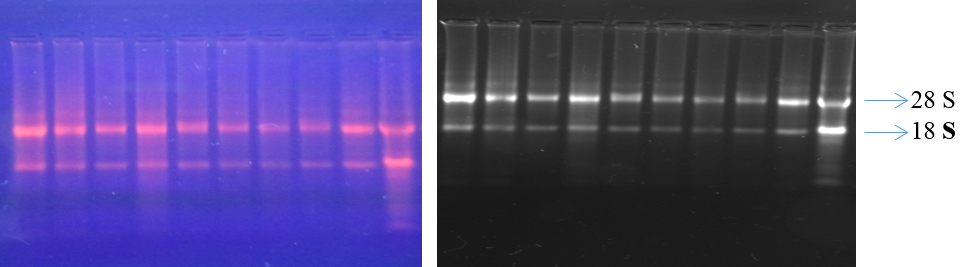

Supplement: Additional file 1: Figure S1. — Ethidium bromide-stained agarose gel samples that showed the extracted colon tissues RNA integrity (1) Seen under UV light. (2) Seen under Lourmat gel documentation system. (TIFF 256 kb) [file 12906_2015_926_MOESM1_ESM.tiff]
